# Erdheim-Chester disease presenting at the central nervous system

**DOI:** 10.4322/acr.2021.321

**Published:** 2021-09-03

**Authors:** Sydney Fasulo, Mina Fransawy Alkomos, Rovena Pjetergjoka, Erinie M Mekheal, Sharon Awasthi, Sahithi Chittamuri, Vinod Kumar, Mehandar Kumar, Amer Akmal, Michael Maroules

**Affiliations:** 1 St. Joseph’s University, Medical Center, Hematology and Oncology Department, Paterson, NJ, USA; 2 St. George’s University, School of Medicine, Grenada, West Indies, Granada; 3 St. Joseph's University, Medical Center, Internal Medicine Department, Paterson, NJ, USA; 4 St. Joseph's University, Medical Center, Pathology Department, Paterson, NJ, USA

**Keywords:** Erdheim-Chester Disease, non-Langerhans cell histiocytosis, and Cerebellar neoplasms

## Abstract

Erdheim-Chester disease is a rare non-Langerhans cell histiocytosis (LCH) that affects different body systems. It was recently recognized as a neoplastic disorder after identifying an activating mutation of the MAPK pathway. Neurological presentations of ECD are rare. We present a case of a 35-year-old male who presented to the emergency department with neck pain, headache and vomiting for 2 months; MRI showed multiple heterogeneous intracranial masses. Neurosurgery performed a suboccipital craniotomy, partially resected the cerebellar mass, and placed a parietal to frontal shunt catheter. Biopsy results from the cerebellar mass demonstrated cerebellar tissue involved by a diffuse proliferation of foamy histiocytes and spindle cells admixed with prominent lymphoplasmacytic infiltrate and positive for CD68, CD163, Factor XIIIa and Fascin. PET scan showed hypermetabolic uptake within the medullary portions of the diffuse abnormal lesions of the distal femurs, tibias, and fibulas, and cardiac MRI was nonsignificant. The patient was started on vemurafenib and continued to show improvement in a 3-month outpatient follow-up.

## INTRODUCTION

Erdheim-Chester disease (ECD) is a rare, systemic non-Langerhans cell histiocytosis (LCH) that affects a diverse group of organ systems, varies in presentation from an insidious onset disease to multi-system organ failure and primarily affects middle-aged adults.^1^ It was recently recognized as a neoplastic disorder after the identification of an activating mutation of the MAPK (RAS-RAF-MEK-ERK) pathway.^1^^,^^2^ The first guidelines were published in 2014 and subsequently included in 2016 WHO classification of tumors of hematopoietic and lymphoid tissues under Histiocytic and dendritic cell neoplasms by the ECD global alliance.^1^^,^^3^

ECD has Skeletal involvement in almost all (> 95%) cases, most often in the long bones of the lower extremities, extra skeletal disease frequent (50 - 60%) in the retroperitoneum, kidneys, cardiovascular system, lung, while central nervous system and pituitary involvement (20 - 30%) and Cutaneous involvement in 25%, but orbit and testis involvement are rare^2^. The most common presenting clinical sign is pain in the long bones and, as ECD affects multiple organ systems, other findings may be central diabetes insipidus, restrictive pericarditis, perinephric fibrosis in addition to sclerotic bone lesions.^1^ Only 3 cases of isolated CNS involvement have been reported previously.^1^^,^^3^^,^^4^ ECD cells have histopathologic features of the xanthogranuloma family and appear as soft tissue infiltrate of bland appearing histiocytes characterized by abundant foamy (xanthomatous) cytoplasm with surrounding fibrosis.^2^^,^^6^^,^^7^ They are immunoreactive for CD68, CD163, factor XIIIa, and fascin and negative for CD1a and CD207, but staining for S100 protein is variable.^1^^,^^6^

## CASE PRESENTATION

We report a case of a 35-year-old Hispanic male with a past medical history of nicotine dependence who presented to the emergency department with the progressive onset of neck pain, headache and multiple episodes of non-bilious non-bloody vomiting for 2 months. He reported that the neck pain was progressive, localized, non-radiating, aggravated by movement, and upon standing from a seated position, along with intermittent vision changes over the past few months. The patient denied fever, chills, night sweats, weight loss, dizziness and his Zubrod performance status was zero.

**Figure 1:**
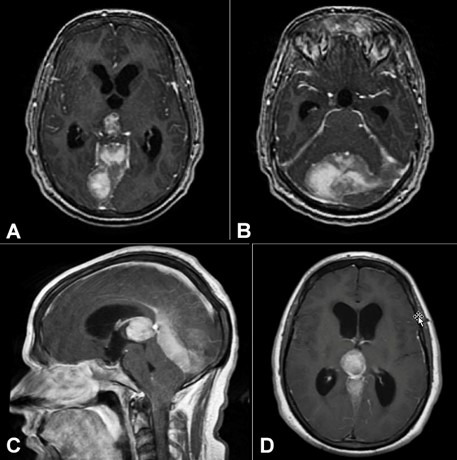
Brain magnetic resonance imaging (MRI). **A** – T1 axillary post-contrast images demonstrate a closely associated mass located parasagittal along the superior portion of the right tentorium and measuring 3.3 x 2.1 x 1.9 cm. Note the mass effect on the right occipital lobe; **B** – The T1 axial post-contrast shows that the enhancing lesion spans the midline within the posterior fossa and appears based within the tentorium and measures 4.5 x 6.1 x 1.8 cm; **C** – The sagittal; and **D** – axial post-contrast T1 isointense shows a heterogeneously enhancing mass of the tectum/pineal gland region, which measures 2.3 x 2.2 x 2.5 cm with pressure effect on the cerebellar hemispheres and tonsillar herniation of approximately 1.8 cm inferior to the cisterna magna.

In the Emergency Department, the patient was vitally stable. The complete blood count and complete metabolic panel were unremarkable, chest x-ray, urine analysis and blood cultures did not show infection. A neurological exam was significant for decreased active range of motion of the cervical spine with pain on palpation of the right trapezius but no appreciable erythema or mass. The exam was negative for focal weakness or paresthesia, and deep tendon reflexes were 2+. Magnetic resonance imaging (MRI) with and without IV contrast showed multiple heterogeneous intracranial masses with resultant mass effect compressing the cerebral aqueduct with dilation of the 3rd and 4th ventricles hydrocephalus and tonsillar herniation (Figure 1).

**Figure 2:**
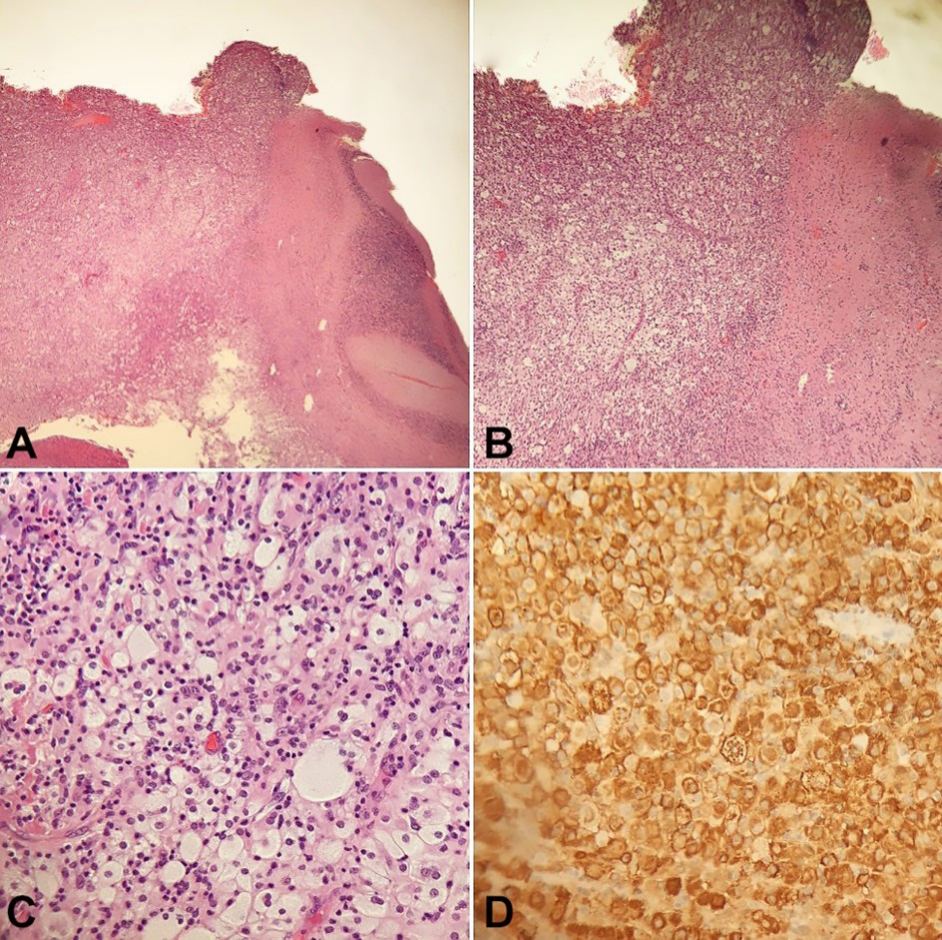
Photomicrographs of the surgical specimen. **A** and **B** – Cerebellar tissue infiltrated by a diffuse proliferation of foamy histiocytes and spindle cells mixed with prominent lymphoplasmacytic infiltrate (H&E, A 40X, and B 100X); **C** – foamy histiocytes and spindle cells (H&E, 400X); **D** – Positive immunoreaction for CD163.

Neurosurgery performed a suboccipital craniotomy, partially resected the cerebellar mass, and placed a parietal to frontal shunt catheter, which significantly reduced the size of the ventricles. Biopsy results from the cerebellar mass demonstrated cerebellar tissue involved by a diffuse proliferation of foamy histiocytes and spindle cells admixed with prominent lymphoplasmacytic infiltrate (Figure 2). Immunohistochemistry showed that the histiocytes were positive for CD68, CD163, Factor XIIIa, and Fascin. They are negative for S100, CD1a and SALL4. Further testing revealed the specimen was positive for BRAF V600E/D mutation, but the assay cannot differentiate between Langerhans and non-Langerhans histiocytosis.

**Figure 3:**
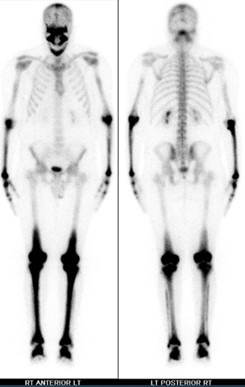
Bone scan shows high-intensity trace uptake in the skull, mandible, predominantly distal long bones of the upper and lower extremities are consistent with widespread histiocytosis.

An evaluation for extracranial involvement was ordered and included a bone scan, PET scan, and cardiac MRI. Bone scan showed high-intensity trace uptake in the skull, mandible, predominantly distal long bones of the upper and lower extremities is consistent with widespread histiocytosis (Figure 3).

PET scan showed hypermetabolic uptake within the medullary portions of the diffuse abnormal lesions of the distal femurs, tibias, and fibulas, and cardiac MRI was unremarkable. The patient was started on vemurafenib and continued to show improvement in a 3-month outpatient follow-up.

## DISCUSSION

The diagnosis of ECD is challenging mainly because of the insidious onset, diverse organ systems affected and non-specific morphological or immunophenotypic characteristics of histiocytes on histopathology. 1^,^^2^^,^^6^It is impossible to differentiate ECD from other non-LCH by imaging and laboratory findings. It is necessary to perform the histological assessment for the definitive diagnosis of ECD.

Goyal et al. 6 present a consensus on the recommendations for diagnosing ECD. It includes; biopsy to confirm xanthomatous histiocytes and to test for BRAF and MAPK-ERK pathway mutations. Next-generation sequencing is the most accurate method; while IHC is less sensitive, but may be used as an initial test. Then, immunohistochemical negative result should be followed by next-generation sequencing; being droplet Digital Polymerase Chain Reaction (ddPCR) the most sensitive. 6In addition to basic laboratory analysis, which includes a complete blood count to evaluate cytopenia, a comprehensive metabolic panel and inflammatory markers, a full-body Positron Emission Tomography-Computed Tomography (PET-CT) scan or a Computed Tomography (CT) with a contrast of chest, abdomen and pelvis. Due to the potential involvement of the cardiac and nervous systems, magnetic resonance imaging (MRI) of the brain with gadolinium and a cardiac MRI should be performed for all patients. Finally, a bone marrow biopsy is necessary to rule out a concomitant myeloid neoplasm.

Most patients with ECD require treatment except for asymptomatic and single organ ECD. Steroids, radiation, and surgery may be utilized as an adjunct therapy to help relieve symptoms.

Treatment for patients with positive BRAF-V600 mutations, cardiac/neurologic disease, or end-organ failure includes vemurafenib, dabrafenib, or encorafenib as the first targeted therapy. MEK-inhibitor may be used for patients without a positive BRAF-V600 mutation. The dosing and duration of treatment have not yet been agreed upon. It has been suggested that treatment should be life-long. Recommended monitoring includes a full-body PET-CT every 2-6 months and organ-specific imaging after initiating therapy and again once stabilization has been achieved. Annual endocrine evaluations are also recommended. 5^,^^6^

Erdheim Chester can be life-threatening with complications such as heart failure, severe damage to the lungs, and kidney failure. However, with treatment, there are patients who are able to live a near-normal life. 2^,^^4^

## CONCLUSION

ECD patients may undergo multiple evaluations and workups, which may delay diagnosis and treatment due to the variation in presentation and the wide variety of organ systems affected.
